# Amyloid Prefibrillar Oligomers: The Surprising Commonalities in Their Structure and Activity

**DOI:** 10.3390/ijms22126435

**Published:** 2021-06-16

**Authors:** Marco Diociaiuti, Roberto Bonanni, Ida Cariati, Claudio Frank, Giovanna D’Arcangelo

**Affiliations:** 1 Centro Nazionale Malattie Rare, Istituto Superiore di Sanità, Viale Regina Elena 299, 00161 Rome, Italy; 2 Department of Systems Medicine, “Tor Vergata” University of Rome, Via Montpellier 1, 00133 Rome, Italy; roberto.bonanni1288@gmail.com (R.B.); giovanna.darcangelo@uniroma2.it (G.D.); 3 PhD in Medical-Surgical Biotechnologies and Translational Medicine, Department of Clinical Sciences and Translational Medicine, “Tor Vergata” University of Rome, Via Montpellier 1, 00133 Rome, Italy; ida.cariati@uniroma2.it; 4 UniCamillus-Saint Camillus International University of Health Sciences, Via di Sant’Alessandro 8, 00131 Rome, Italy; claudio.frank@unicamillus.org; 5 Centre of Space Bio-Medicine, “Tor Vergata” University of Rome, Via Montpellier 1, 00133 Rome, Italy

**Keywords:** amyloid, neurodegeneration, prefibrillar oligomers, structure, membrane permeabilization, Ca^2+^ influx, neurotoxicity, synaptic transmission, long-term potentiation

## Abstract

It has been proposed that a “common core” of pathologic pathways exists for the large family of amyloid-associated neurodegenerations, including Alzheimer’s, Parkinson’s, type II diabetes and Creutzfeldt–Jacob’s Disease. Aggregates of the involved proteins, independently from their primary sequence, induced neuron membrane permeabilization able to trigger an abnormal Ca^2+^ influx leading to synaptotoxicity, resulting in reduced expression of synaptic proteins and impaired synaptic transmission. Emerging evidence is now focusing on low-molecular-weight prefibrillar oligomers (PFOs), which mimic bacterial pore-forming toxins that form well-ordered oligomeric membrane-spanning pores. At the same time, the neuron membrane composition and its chemical microenvironment seem to play a pivotal role. In fact, the brain of AD patients contains increased fractions of anionic lipids able to favor cationic influx. However, up to now the existence of a specific “common structure” of the toxic aggregate, and a “common mechanism” by which it induces neuronal damage, synaptotoxicity and impaired synaptic transmission, is still an open hypothesis. In this review, we gathered information concerning this hypothesis, focusing on the proteins linked to several amyloid diseases. We noted commonalities in their structure and membrane activity, and their ability to induce Ca^2+^ influx, neurotoxicity, synaptotoxicity and impaired synaptic transmission.

## 1. Introduction

Amyloid proteins are a large family of proteins with the common tendency to aggregate through a process that, triggered by a misfolding event, involves first a slow and thermodynamically unfavorable nucleation phase followed by a rapid elongation phase (seeding-nucleation model) leading to the formation of mature fibers (MFs) [1,2]. The misfolding process can involve practically all proteins and has therefore been defined as their “dark side” [3].

Several highly diffused or rare human diseases are linked to the formation of amyloid aggregates through the misfolding process of the involved proteins. The most famous include amyloid-β (Aβ), α-synuclein (α-syn), amylin (hIAPP) and prion (Pr), which are linked to Alzheimer’s disease (AD), Parkinson’s disease (PD), type II diabetes and Creutzfeldt–Jacob’s Disease (C–JD), respectively [1,4]. Chiti and Dobson took stock of this topic in a recent, important and exhaustive review [1].

During the common aggregation process, which occurs both in vitro and in vivo, low-molecular-weight prefibrillar oligomers (PFOs) appear before MFs, rich in cross-β-sheets. PFOs can further aggregate with each other to form annular or linear protofibrils (APFs or LPFs) and, finally, MFs. Up to now, PFOs have been considered the most toxic species, responsible for cellular damage and subsequent amyloid toxicity [1,5,6,7,8,9].

A correlation between PFOs and a group of cognitive disorders involving the central nervous system (CNS) and known as “neurodegenerative diseases” has been demonstrated [10,11,12]. The most known and widespread of these diseases is undoubtedly AD, characterized by the extracellular accumulation of Aβ protein deposits and intraneuronal tangles of the hyperphosphorylated tau protein [13]. In PD, which is the second most common neurodegeneration, aggregates of the α-syn protein (Lewy bodies) accumulate in dopaminergic neurons of the substantia nigra [14]. Again, in Huntington’s disease (HD), an expansion of the CAG repeat is the cause of the presence of a polyglutamine tract (Ploy-Q) in the huntingtin protein (Htt), which promotes its aggregation [15]. Moreover, misfolded prion protein (PrP) aggregates are responsible for a group of conditions known as prion diseases [16].

Although the symptomatology and epidemiology of these diseases have been extensively characterized [17], the molecular mechanism by which oligomeric aggregates induce neurotoxicity and cell death has not been fully elucidated. This is mainly due to the rapid rate and heterogeneous nature of the aggregation process, which amplifies the difficulty scientists have in identifying the amyloid aggregates responsible for toxicity [18,19]. As recently pointed out by Benilova et al., the tendency of proteins, such as Aβ, to rapidly aggregate during experiments leads to a difficult and uncertain identification of the structure responsible for a well-defined biologic effect [18]. For this reason, amyloid experimental models are also used, i.e., proteins, which although not associated with any pathology, undergo an aggregation process and exert typical neurotoxic effects both in vitro and in vivo.

Notably, several studies of both amyloid proteins and amyloid models have suggested that oligomers, regardless of the nature of the protein from which they derive, share a common structure and action mechanism [11,19,20,21]. It has been reported that a common feature of all neurodegenerative diseases is synaptotoxicity, resulting in reduced expression of synaptic proteins and impaired synaptic transmission by neurotoxic oligomeric aggregates [22,23,24,25]. Recently, Soto and Pritzkow provided a critical discussion on the role of protein misfolding and aggregation in neurodegeneration. They highlighted commonalities and differences between distinct protein aggregates and discussed evidence supporting the hypothesis that misfolded aggregates may be transmissible by the prion principle following a “cross seeding” behavior [26].

To further investigate this intriguing hypothesis of the existence of a common structure and action mechanism in the amyloid neurodegenerations, in the present review we will focus our attention on i) commonalities between structural and functional features of the toxic PFOs of amyloid proteins and models and ii) commonalities between PFO-induced effects on synaptic function and transmission, considered as the main molecular and electrophysiological mechanisms responsible for neuronal dysfunction related to cognitive impairment.

## 2. Structural Commonalities

As argued by Glabe in 2006, soluble spherical aggregates of about 3–10 nm in diameter have been observed for many types of amyloid proteins by several microscopy techniques, and have been called micelles, PFOs, protofibrils and Aβ-derived diffusible ligands (ADDLs) [20]. Our interest will be focused on PFOs, which are the intermediate species formed during the aggregation process and are considered the most toxic species.

In general, PFOs are defined as neurotoxic and soluble low-molecular-weight aggregates, spherical shaped with diameters in the order of nanometers, mainly in random configurations, different from the β-sheet configuration of amyloid fibres.

In a pivotal paper in 2003, Kayed et al., reported the production of an antibody that specifically recognizes micellar Aβ and not soluble, low-molecular-weight aggregates (low-MW) or fibrils. Notably, this antibody also specifically recognizes soluble oligomers among all other types of amyloidogenic proteins and peptides examined, including α-syn, hIAPP, polyglutamine, lysozyme, human insulin and PrP(106–126) [11]. Most important, this antibody was able to inhibit toxicity mediated by all other types of soluble oligomers. Thus, the authors concluded that the soluble oligomeric forms of all tested amyloids share a common structure that can mediate toxicity by a common mechanism. Their conformation is different from that of soluble monomers, low-MW oligomers and fibrils; in addition, the marked epitope was formed from a specific conformation of the polypeptide backbone and was independent from the amino acid side chain in this region. With this antibody, it was possible to discriminate between two fundamental distinct types of amyloid oligomers: prefibrillar and fibrillar [11,27].

Furthermore, in 2008, Yoshiike et al., observed that the A11 antibody is also able to react with several purified human native heat-shock proteins, such as Hsp27, 40, 70 and 90, as well as yeast Hsp104, bovine Hsp70 and the bacterial chaperone GroEL. Nevertheless, the authors reported that the binding specificity between the A11 antibody and oligomers, as indicated by Kayed et al., exists and is conformation specific rather than sequence specific [28]. Notably, the authors reported that the A11 antibody specifically binds oligomeric intermediates and not misfolded monomers or amylogenic fibers, emphasizing the existence of a generic conformation common to all oligomeric intermediates formed by a wide variety of amyloid proteins. We note that they reported that the ability of A11 to react with native heat-shock proteins indicates only their occurrence in the monomeric native configuration of these proteins of the conformation identified in the amyloid oligomers. Measuring their molecular weight, which indicates they are not monomers, can identify PFOs.

Common features of cytotoxic amyloid species have been also described in 2010 by Bolognesi et al. for several proteins, such as wild-type Aβ_1-42_, the I59T variant of human lysozyme and an SH3 domain. Their results suggest a model in which the exposure of the hydrophobic surfaces, as a result of the aggregation of misfolded species, is a crucial and common feature of these pathogenic species [29]. These data strongly support the hypothesis that the exposure of hydrophobic patches is a key feature of the toxicity of extracellular oligomeric species. In the so-called “coalescence and reorganization” model, the generation of a stable hydrogen-bonded core drives the exposure of the hydrophobic residues that were buried in the initial collapse [30,31].

### 2.1. Proteins Linked to Neurodegenerative Diseases

Aβ

In 1994, Soreghan et al., described the formation of micelles in solution by Aβ peptides [32] while, in 1996, another group featured spheroidal structures by atomic force microscopy (AFM) and called them “protofibrils” [33]. Aβ PFOs were observed to vary in size from tetramers to 20-mers while biggest oligomers, such as ADDLs, globulomers and protofibrils, were classified as fibrillar [34].

Most scientific evidence agrees that the PFOs’ formation is accompanied by a conformational change in the protein structure, mainly consisting of an increased content of β-sheet [35,36,37]. As an example, Fourier-transform infrared spectroscopy (FTIR) studies have suggested a characteristic antiparallel β-structure for Aβ oligomers [38]. Furthermore, a model has been proposed in which four Aβ_42_ molecules, each consisting of three β-strands with a head-to-tail orientation, form a β-sheet and four of these sheets are stacked in the prefibrillar oligomers [39].

In 2006, Lesné et al., used transgenic mice Tg2576 to demonstrate that memory defects occur in “mild-aged” mice without neuronal loss, while abundant neuritic plaques containing Aβ_42_ were observed in older mice. In addition, in “mild-aged” mice they found an extracellular accumulation of 56 KDa soluble amyloid assemblies, named Aβ*56 and corresponding to a dodecamer. Therefore, the authors concluded that this aggregate impairs memory by an unknown mechanism, independent of either plaque formation or neuronal loss [40]. The dodecamer, rather than fibrils, was also indicated as a determinant of AD by Cheng et al., in a study of transgenic mice and the Artic mutant alloform of Aβ_42_ [41]. Moreover, Bernstein et al., focused on the importance of tetramers and dodecamers in in vitro studies [42,43].

In 2009, Ono and colleagues investigated the relationship between oligomer structure and toxicity using a combination of structural and biological techniques. In detail, by preparing and studying specific stabilized oligomers, fractionated in pure form, the authors found that Aβ monomers were largely unstructured, as opposed to oligomers that showed order-dependent increases in β-sheet content. They observed that the neurotoxic activity increased disproportionally with oligomer order: dimers were three times more toxic than monomers, while tetramers were 13 times more toxic [44].

The desire to elucidate the structure and properties of Aβ PFOs has a long history and techniques for characterizing their protein structure are now numerous [45]. However, due to their small size (<10 nm), heterogeneity, metastability, not-crystalline nature of the prefibrillar oligomers and different protocols used to prepare samples, there are so far many papers on their general composition and, despite many efforts in this area, only few studies have provided atomic structures for Aβ PFOs [18,46,47,48,49].

In 2016, Serra-Batiste et al., studying the formation of Aβ PFOs within a membrane environment, observed that the two species Aβ_40_ and Aβ_42_ showed different behavior. Specifically, Aβ_40_ aggregated into amyloid fibrils unable to penetrate the bilayer, whereas Aβ_42_ assembled into oligomers able to form well-defined pores in the membrane. Notably, only Aβ_42_ oligomers adopted a specific structure characterized by the β-barrel arrangement, called β-barrel pore-forming Aβ_42_ (βPFOs_Aβ42_) [50].

Recently, Ciutad et al., by nuclear magnetic resonance (NMR) spectroscopy and mass spectrometry (MS), better defined the 3D atomic structures of βPFOs_Aβ42_. In particular, the authors presented the structure of a tetramer that includes a six-stranded β-sheet core: the two faces of the β-sheet core are hydrophobic and surrounded by the membrane-mimicking environment, while the edges are hydrophilic and solvent-exposed. As the concentration of Aβ_42_ in the sample increases, octamers formed from two tetramers facing each other form a β-sandwich structure. Notably, Aβ_42_ tetramers and octamers inserted into lipid bilayers as well-defined pores, classifying them as potentially responsible for the strong toxicity typical of AD [51].

**Figure 1:**
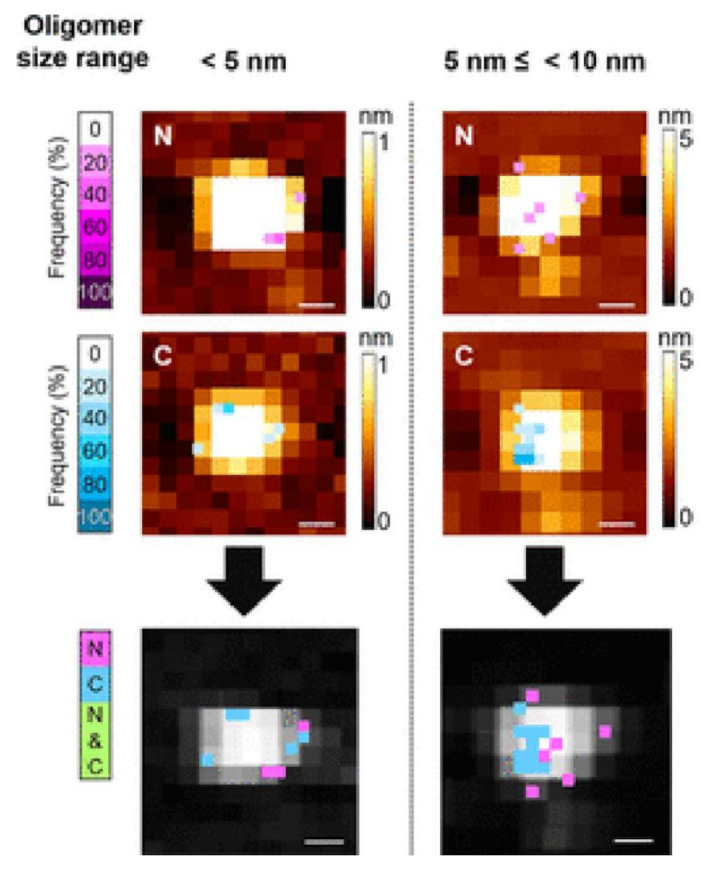
The surface structure of a PFO. Yoon et al., studied, at molecular resolution, the structure of individual Aβ PFOs by means of an innovative AFM technique, which involves the use of tips linked to the specific terminal antibody. Flexible zones, consisting of N- and C-terminals exposed to the solvent, emerge from a rigid and hydrophobic core. Bar represents 10 nm (Reprinted with permission from Yoon et al. [52]. Copyright 2019 American Chemical Society).

Thus, the hydrophobic profile of the PFOs’ surface seems to drive their interaction with cellular targets. Remarkably, for the first time in our knowledge, Yoon et al., studied the surface structure of individual Aβ oligomers. They were able to visualize their surface at atomic resolution using an innovative AFM technique, which involves the use of terminus-specific antibody-tethered tips. The authors mapped the N- and C-terminus surface distributions and the elastic modulus of individual oligomers less than 10 nm in diameter (Figure 1), finding two types of regions: one flexible, where both N- and/or C-terminals were recognized, and a stiff one where they were not. Moreover, using a specific antibody for the hydrophobic Aβ_17-21_ fragment, they showed that this sequence was not accessible anywhere on the surface, concluding that residues Aβ_17-21_ together with Aβ_30-35_ formed the hydrophobic oligomer core by inter-peptide contacts. Interestingly, they discussed that part of the hydrophobic C-terminus was partially buried at the core, while the end regions were exposed on the surface [52].

α-Syn

It has been reported that certain mutations in human α-syn are associated with familial forms of early-onset PD and that in these mutants there is a rapid oligomerization of the protein, suggesting a correlation between oligomeric structures and neurotoxicity [53]. Furthermore, FTIR studies have shown that α-syn oligomers adopt an antiparallel β-sheet structure, in contrast to the fibril in which a parallel β-sheet arrangement was observed [54]. The morphology of α-syn oligomers was also investigated by AFM and AFM-infrared spectroscopy (AFM-IR), detecting a typical spherical shape with diameter and thickness larger than those of the monomers [55,56].

Fusco et al., recently defined the structural basis of cellular toxicity exerted by α-syn PFOs, investigating two types of stabilized oligomers with considerably different toxicity [57]. It was observed that the toxic oligomer was able to induce an increase in intracellular reactive oxygen species (ROS) and a reduction in mitochondrial activity in neuronal cells. Furthermore, by solid-state MNR (ssNMR) they described for the first time the oligomer molecular structure, observing that the toxic oligomer had a rigid core rich in β-sheet, absent in the core of the non-toxic one. FTIR spectroscopy showed that in the toxic oligomer β-sheet and random content coexisted, while in the non-toxic oligomer the protein was in random configuration [57]. They showed that, in the toxic PFO, the N-terminus of α-syn was accessible and highly dynamic, whereas, in the non-toxic one, it was less dynamic and located in the core. The C-terminal region of α-syn was highly dynamic in both types of oligomer. Notably, the toxic PFO was able to permeabilize lipid membranes 10 times more than the non-toxic one, penetrating the hydrophobic interior of the bilayer driven by the exposed N-terminal region and leaving outside its dynamic regions. The authors concluded that toxicity was determined by two structural elements: i) an exposed highly lipophilic region of the protein that promotes a strong interaction with the membrane surface and ii) a rigid oligomeric core rich in β-sheet capable of inserting in the lipid bilayer and disrupting the membrane integrity [57]. Thus, the surface accessibility of the highly flexible N-terminal region of α-syn is a key determinant of PFOs’ toxicity.

Tau

In 2007, Maeda et al., providing AFM images of a tau solution incubated for 72 h, revealed the presence of spherical and elliptical granules and fibrils, thus suggesting that tau oligomers enriched in β-sheet conformation might be composed of partially folded monomers, different from native monomers. The authors also reported that, at sizes above 20 nm, the formation of tau fibrils is promoted [58]. Furthermore, Flach and colleagues showed electron micrographs representing tau oligomers, which appear as spherical structures with diameters of less than 12 nm [59].

Recently, it has been shown that, as in other amyloid species, the granular oligomers of the tau protein, which vary in size from 5 to 50 nm, show a characteristic spread that increases their neurotoxic effect [60]. Moreover, Karikari et al., used circular dichroism (CD) spectroscopy to study the secondary structure content of the C291R variant of the tau protein, suggesting that it is characterized by both the presence of an aggregation-promoting fragment and a greater propensity to adopt a β-sheet conformation [61]. Notably, transmission electron microscopy (TEM) analysis revealed that the presence of the C291R mutation favors the formation of non-fibrillar oligomers rather than fibrils [61].

Htt

AFM analysis determined that the size of globular oligomers isolated from brains of HD mouse models (R6/2 and HdhQ150) is identical to that of oligomers produced by in vitro aggregation of recombinant exon 1 proteins from the Htt gene [62]. Magic-angle spinning (MAS) and ssNMR techniques were used to investigate the Htt fibrillar structure, detecting the presence of β-sheets containing intermolecular β-hairpin [63]. It was also observed that the presence of mutations determining β-hairpin formation in poly-Q peptides increases the aggregation rate in structures with an antiparallel β-sheet architecture [64]. Again, mutations that increase the length of the poly-Q tract in exon 1 of the Htt gene (MHtt) have been associated with the formation of oligomers that further aggregate into large insoluble structures characterized by an increased β-sheet secondary structure content [65].

Prefibrillar Htt oligomers isolated from human or mouse HD brain samples may promote amyloid polyglutamine formation; indeed, immunopurification of misfolded Htt, performed on brain homogenates with an antibody capable of recognizing Aβ, α-syn and Htt oligomers, showed the presence of active amyloid seeds [66].

PrP

Soluble aggregates of misfolded prion protein, rich in β-sheet and slightly resistant to the action of proteinase K, have been indicated as key intermediates in the pathogenesis of prion diseases, as they are extremely neurotoxic both in vivo and in vitro [67,68].

Although the structure of PrP^C^ is characterized by a single small two-stranded β-sheet, a predominance of β-structures and a typical aggregative behavior can be observed in the PrP^Sc^ protein, leading to the formation of oligomers and then amorphous aggregates and amyloid fibrils [69].

In 2011, Bjorndahl et al., reported that the formation of a soluble β-sheet-rich intermediate (PrP^β^), which precedes the appearance of fibrils, is favored at acidic pH. Specifically, dynamic light scattering (DLS) data revealed that the diameter of the monomeric protein remains stable in a pH range between 3 and 5 while it increases at lower pH, indicating protein oligomerization [70]. At pH 1, the diameter of the aggregate reached a size of 18-19 nm and a molecular weight of 628 KDa, corresponding to an oligomer consisting of approximately 30 monomers. Furthermore, through CD analysis, the authors observed that pH changes had an impact on the secondary structure content, as at low pH there was the reduction in α-helical structures and the increase in β-sheets [70].

An eight-amino-acid duplication in the hydrophobic region of PrP^C^ (early-onset allele, HRdup), which is responsible for the onset of Gerstmann–Sträussler–Scheinker disease, appears to result in the appearance of the β-structure in this region [71,72].

### 2.2. Proteins Non-Linked to Neurodegenerative Diseases

The rapid rate and heterogeneous nature of the aggregation process of the main amyloid proteins makes it extremely difficult to identify the aggregate responsible for toxicity [18,19]. For this reason, amyloid models are used, i.e., proteins characterized by the typical aggregation behavior and that form oligomers able to exert toxic effects, both in vitro and in vivo, comparable to that exerted by disease-correlated amyloid proteins.

HEWL

An example is hen egg white lysozyme (HEWL), a normally harmless protein able to aggregate, forming roughly spherical β-sheet-rich oligomers that appear to convert over time into mature amyloid protofibrils and fibrils [73].

As early as 2011, it was reported that HEWL oligomers are structures with flexibility and hydrophobicity, allowing them to interact with mitochondrial membranes [74]. Subsequently, through FTIR spectroscopy, Zou et al., demonstrated that HEWL can form fibrils and oligomers with distinct β configurations. In particular, they observed that, at high temperatures, the formation of fibrils with a parallel β-sheet configuration is promoted, whereas at room temperature, the formation of oligomers with an anti-parallel β-sheet configuration is favored [75].

Recently, Verma et al., showed that the aggregation process of HEWL is also affected by the presence of arsenic trioxide, a metal pollutant. Here, the authors observed that the initial phase of the aggregation process depends on the exposure of hydrophobic surfaces that tend to organize into β-sheet structures [76].

Insulin

The ability to form amyloid aggregates has also been documented for exogenous insulin, which is used as a therapeutic agent for diabetes and appears to form insoluble deposits containing amyloid fibrillar structures near the site of administration [77]. Recently, Ratha et al., characterized, at high-resolution, the structure of a partially folded intermediate of insulin, suggesting that this state may represent the starting point of the aggregation process. The authors observed slight structural changes in this intermediate compared to the monomer, claiming that these changes lead to the formation of a hydrophobic cavity in the center of the protein that acts as a nucleation center for oligomer formation [78].

Furthermore, using surface-enhanced Raman spectroscopy (SERS), Kurousky et al., found that, at the stage of the aggregation process preceding fibril formation, the amount of insulin oligomers increases significantly over a relatively short period of time and then slowly decreases [79] This observation, in addition to providing information about the structural organization of oligomers over time, is further evidence to support insulin’s ability to form oligomers that aggregate into fibrillar structures. This was also confirmed by Dolui et al., who observed, during the process of insulin aggregation, the formation of spherical oligomers of 3–5 nm that organize into fibrils approximately 5–8 nm thick [80].

Sup35

Another example of an amyloid model is Sup35, an essential protein that acts as a translation termination factor in *Saccharomyces cerevisiae* [81]. It is known that the N-terminal portion of Sup35 is a glutamine/asparagine-rich domain that has a high propensity to form fibrils in vitro [82,83]. Studies on the yeast prion [PSI+] revealed an extensive and compact β-sheet structure in Sup35 aggregates [84].

In 2011, Liu et al., reported that the N-terminal and central region of Sup35 can assemble and promote the formation of prefibrillar granular aggregates of 2–5 nm in diameter [85]. Recently, Konno et al., used high-speed atomic force microscopy (HS-AFM) to study the formation of Sup35 oligomers. After an incubation period of 30 min, the authors observed the formation of particles with a height of 1.7 nm, which were taller than those of the monomer, suggesting that they were oligomers. It is noteworthy that the height of these structures gradually increased during the aggregation process, eventually reaching platoon at around 3–4 nm [86].

PI3-SH3

The SH3 domain of bovine phosphatidyl-inositol-3′-kinase (PI3-SH3) is another example of small globular proteins that, under appropriate conditions, can form fibrillar aggregates and exert a potent cytotoxic effect in vitro [87].

Bolognesi et al., studied the SH3 domain, together with the wild-type Aβ_1-42_, its Arctic variant and the I59T variant of human lysozyme, showing that it shares the common features of cytotoxic amyloid species: the formation of a stable hydrogen-bonded oligomer core that drives the exposure of the hydrophobic residues, responsible for its toxicity [29].

HypF-N

The 91-residue N-terminal domain of the *Escherichia coli* HypF protein (HypF-N) is another valuable model for investigating the structure of toxic amyloid PFOs.

As described by Campioni et al., in 2010, HypF-N is able to form oligomers that are sufficiently stable to maintain their structure even when transferred to conditions that are different from those that promote their formation [88,89]. The authors obtained information about the structure of a toxic HypF-N PFO of about 7 nm, characterized by a β-sheet core where the hydrophobic regions of the proteins were only partially organized and solvent-exposed. Notably, they concluded that the HypF-N PFO toxicity depends on the extent to which hydrophobic residues were flexible and exposed on the oligomer surface and that these properties are at the origin of the pathogenesis of important debilitating human diseases.

sCT

Since 1993 it has been shown that salmon calcitonin (sCT) can aggregate and be toxic to cells in culture, although it is not directly involved in any neurodegenerative diseases [90]. More recently, we have successfully proposed sCT as an amyloid model in the study of the molecular mechanisms at the basis of neurotoxicity of the large family of amyloid proteins due to its very slow aggregation rate, allowing us to follow the process from the early stages, without fixing procedures that can influence the oligomer structures [91]. In 2014, we identified the most neurotoxic species among all aggregates, which are the PFOs. They were purified by size exclusion chromatography (SEC) and studied by SDS-PAGE, TEM and CD spectroscopy. We found metastable sCT tetramers, forming small globules of about 9 nm in diameter with the protein mainly in random configuration (72%) and with an ordinated core with the protein in β-sheet (about 20%) [92]. In a following paper, we showed that only those PFOs were able to permeabilize lipid bilayers thanks to their external, flexible, disordered and charged shell and the internal hydrophobic core [93].

Notably, all our findings are in line with those reported for Aβ, α-syn e HypF-N and others [52,57,88].

## 3. Functional Commonalities

We present papers where authors described similar effects induced by exogenous and endogenous PFOs. When PFOs were prepared and administered exogenously, the authors described their preparation protocol and structures were in agreement with the wide definition of PFOs (specified in the Introduction). In the case of papers where authors presented effects induced by endogenous PFOs, they indicated and documented the presence of oligomers, not monomers and fibers, purified and characterized in several ways. We only compared the effects induced by several kinds of the exogenous administrated PFOs and those observed in mouse models of neurodegenerative diseases, highlighting “commonalities”.

Although PFOs share several commonalities, such as toxicity, propagation, tendency to self-assemble and synaptotoxicity, in this section we will consider only some of these aspects. In particular, we will focus on the molecular mechanisms leading to their neurotoxicity and on their effects on synaptic structure and function.

### 3.1. Neurotoxicity Molecular Models

It has been suggested that the different types of oligomers, as they present similar structural features, could share a common mechanism of neurotoxicity. This neurotoxic action could be expressed through the deregulation of Ca^2+^ homeostasis and cell death [11,94]. It has been also reported that the lipid composition of membranes may modulate the cellular response to the toxic action of amyloid aggregates, thus explaining the different vulnerability of different cell types to amyloid species [95]. In particular, the importance of GM1, a major component of “lipid-rafts”, in the cellular membranes [96] as a mediator of amyloid toxicity has been widely documented [93,96,97,98].

**Figure 2:**
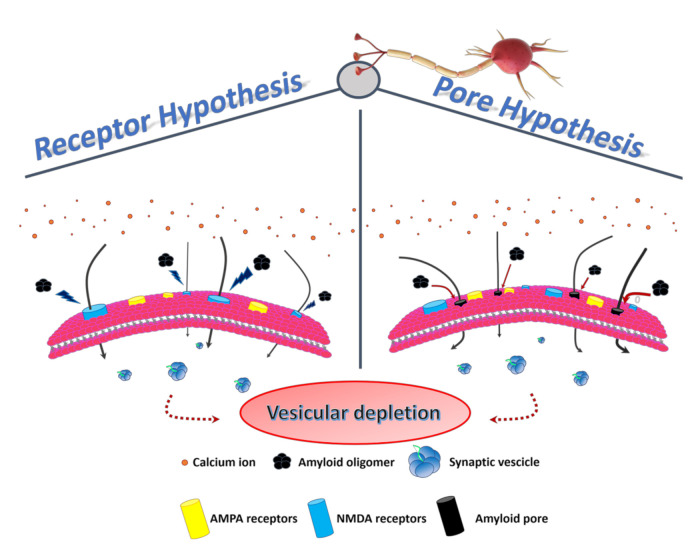
Membrane permeability and vesicular depletion. In the physiological condition, membrane receptors regulate the entry of Ca^2+^ into the cell, ensuring the preservation of the pool of vesicles containing the neurotransmitter. In the pathological condition, characterized by the unbalance of the Ca^2+^ influx, two hypotheses have been formulated to explain the altered membrane permeability: in the first, PFOs directly activate NMDA receptors (receptor hypothesis), while in the second, PFOs form pores (continuous red arrows) in the membrane (pore hypothesis). Increased Ca^2+^ influx (black arrows) results in vesicular depletion (dashed red arrows) and consequent cell death.

Kayed and Lasagna-Reeves, in 2013, reviewed the molecular mechanisms proposed for amyloid oligomer toxicity. Two kinds of mechanisms were proposed: “receptor-mediated” and “cellular membrane”. In the first, oligomers disrupt Ca^2+^ homeostasis, interacting with several cellular receptors, while in the second, forming ionic channels in the membrane [12]. In any case, the intracellular [Ca^2+^] rise seems to play a fundamental role in both paradigms (Figure 2).

Concerning “receptor-mediated” toxicity, several receptors have been considered to play a key role such as nerve growth factor or the N-methyl-D-aspartate (NMDA) receptors, which are a subtype of ionotropic glutamate receptors responsible for Ca^2+^ regulation [25,93,99,100,101,102,103]. However, in their review, Kayed and Lasagna-Reeves concluded that a major unknown was the identity of the receptor that bind oligomers and mediates neuronal dysfunction, and some studies are contradictory [12].

Concerning “cellular membrane” toxicity, in 2002 Kourie and Henry hypothesized that protein misfolding that leads to the exposure of hydrophobic regions of amyloid proteins renders them potentially cytotoxic. Amyloid proteins, such as PrP, Aβ, amylin (islet amyloid polypeptide—IAPP) and calcitonin (CT), interact with membranes, inducing damage via the formation of ion channels, the so-called “amyloid pore hypothesis” [104]. In particular, Kayed et al., in 2004 showed that only soluble oligomers, and not MFs, from several types of amyloids (Aβ, α-syn, IAPP, polyglutamine and PrP) specifically increased the membrane conductance of planar lipid bilayers, regardless of the protein sequence [94] and, in 2005, Quist et al., directly visualized by atomic force microscopy (AFM) typical amyloid pores for a group of six amyloid proteins [105]. The “amyloid pore hypothesis” was supported by Kagan et al., in 2004, who reported on the physiologic effects induced by this phenomenon, including Ca^2+^ dysregulation, membrane depolarization, mitochondrial dysfunction, inhibition of long-term potentiation (LTP) and cytotoxicity [106].

Lashuel and Lansbury summarized in 2006 the existing supportive circumstantial evidence about several neurologic amyloid diseases, including AD, PD, British dementia, amyotrophic lateral sclerosis (ALS), C–JD and Huntington’s disease. They speculated about the hypothesis that they were all caused by protein aggregates that mimic bacterial pore-forming toxins, which in general form well-ordered oligomeric membrane-spanning pores able to kill neurons [107]. 

Recently, Angelova and Abramov noted that α-syn and Aβ in an oligomeric state, although with different primary targets, share a common mechanism that involves formation of pore-like structures in the plasma membrane, consequent Ca^2+^ homeostasis dysregulation, mitochondrial dysfunction and oxidative damage [108].

Finally, in 2019, Yasumoto et al., in an important and extensive paper, clearly demonstrated that Aβ oligomers disturbed membrane integrity, concluding that “membrane pore formation may also impair cellular and synaptic functions and is consistent with the observed loss of membrane integrity demonstrated by MTT, LDH, and calcein/ethidium homodimer-1 assay, [Ca^2+^] elevation, resting membrane potential increase input resistance decrease and LTP impairment” [109].

Soluble amyloid oligomers have a neurotoxic effect even when formed from proteins not directly related to neurodegenerative diseases, used as amyloid models [94,110,111]. In 2019, using salmon calcitonin (sCT) as an amyloid model due to its slow aggregation rate, allowing us to prepare stable samples without photochemical cross-linking, we tested the effects of native sCT PFO-enriched solutions in primary neurons and mice brain slices in terms of Ca^2+^ influx, cellular viability, LTP impairment, post-synaptic densities and synaptophysin expression. The results indicate that PFO-enriched solutions induced abnormal Ca^2+^ influx, which could only in part be ascribed to the NMDAR activation. Thus, we proposed an innovative “unified” neurotoxicity mechanism where both paradigms coexist, where the membrane permeabilization per se is not able to induce neurotoxicity but triggers an abnormal activation of the NMDAR [25].

Notably, in this paper, we obtained, by several techniques, experimental results very similar to those published by Yasumoto et al., for Aβ [109]. This similarity prompts the intriguing hypothesis in the open debate about the existence of a “common mechanism” in the pathogenesis of amyloid neurodegenerations.

#### 3.1.1. Proteins Linked to Neurodegenerative Diseases

Aβ

In 2005, Demuro et al., evaluated the increase in intracellular Ca^2+^ levels in SH-SY5Y cells treated with monomers, oligomers and fibrils of the Aβ_42_ protein. Following extracellular application of monomers or fibrils, the authors observed no change in intracellular Ca^2+^, whereas an equal amount of oligomer generated large and rapid increases in Ca^2+^-dependent fluorescence. Notably, the authors showed that the increase in intracellular Ca^2+^ levels depended, for the most part, on an extracellular influx, while only a small part could result from intracellular Ca^2+^ release. The authors proposed that Aβ_42_ oligomers can dramatically increase membrane permeability and subsequently penetrate into cells and affect intracellular membranes, causing an imbalance in Ca^2+^ homeostasis and probably also of high ions [112].

In 2010, Cizas et al., observed a striking correlation between the toxicity of Aβ_42_ oligomers, assessed on neuronal cells by staining with propidium iodide and Hoechst, and aggregate size. In particular, they observed that oligomers consisting of more than 14 units did not cause significant cell death, whereas the cytotoxicity of smaller oligomeric species could lead to a strong reduction in cell survival in cells exposed to treatment [113].

In 2014, Sepúlveda et al., reported that Aβ oligomers interact with the neuronal membrane where they induce the formation of pores leading to the influx of Ca^2+^ ions and increased release of synaptic vesicles, resulting in synaptic impairment and vesicle depletion [114]. In the same year, Kim et al., observed that an oligomeric form of synthetic Aβ_42_ was able to induce HT22 cell death, through the involvement of the apoptotic protein BAK and the formation of pores in vesicular membranes. Indeed, suppression of the BAK protein by short-interfering RNA promotes cell survival and prevents the release of cytochrome C from the mitochondria, suggesting that Aβ oligomers can induce cell death by activating the BAK-dependent apoptotic pathway [115].

In 2016, Peters et al., evaluated the effects of some representative Aβ peptide fragments on the cell viability of primary hippocampal neurons by MTT viability assay, concluding that, although Aβ_1-42_ oligomers are the most cytotoxic species, Aβ_1-28_, Aβ_25-35_, and Aβ_17-42_ oligomers were also capable of significantly reducing cell viability [116]. Contextually, they detected an increase in intracellular Ca^2+^ levels induced by the different Aβ oligomers, suggesting the hypothesis of amyloid pore formation as a mechanism responsible for neurotoxicity [116].

Finally, in 2017 Kandel et al., attempted to characterize the interaction of Aβ_25-35_ oligomers with anionic membranes by observing a significant influx of intracellular Ca^2+^ because of pore formation in the membrane. Notably, the authors reported that reducing the anionic charge of membranes significantly reduced the binding of Aβ oligomers and the subsequent pore formation, highlighting the importance of electrostatic interactions in the mechanism of action of Aβ_25-35_ oligomers [117].

α-syn

In 2007, Danzer et al., evaluated the cytotoxicity effects of different types of α-syn oligomers on SH-SY5Y cells. Following exposure of the cells to oligomers, they measured intracellular Ca^2+^ levels and seeding capacity. The authors reported that oligomeric α-syn species were not only able to form pores in synthetic membranes, but also to increase Ca^2+^-dependent fluorescence in SH-SY5Y cells. Furthermore, it was observed that the increase in intracellular Ca^2+^ was dependent on extracellular conditions. In fact, in SH-SY5Y the Ca^2+^ increase was also detected after blocking a non-specific Ca^2+^ channel with cobalt and only when the extracellular phosphate buffer contained Ca^2+^. Interestingly, the authors also measured the membrane potential of primary cortical neurons to see if Ca^2+^ influx was indeed caused by pore formation and detected a depolarization of the potential [118].

In 2011, Winner et al., demonstrated the toxicity of α-syn oligomers in vivo by testing different variants of the protein. Specifically, in addition to observing the formation of pore-like structures in vitro, the authors reported that recombinant α-syn variants promoting oligomer formation induced the loss of dopaminergic cells in the rat. Furthermore, immunoblot analysis performed on rat brain tissue treated with the oligomerization-promoting protein variants showed an abundance of α-syn trimmers, compared to animals treated with wild-type α-syn. The efficacy of these variants in promoting the formation of highly cytotoxic oligomeric aggregates was also tested in a cellular system, where they induced Ca^2+^ influx and cell death [119].

More recently, Yang et al., provided further evidence of the toxicity of α-syn oligomers (O-α-syn) in vitro. The authors found that O-α-syn strongly reduced the amount of tyrosine hydroxylase (TH)-labelled dopaminergic neurons in the substantia nigra. Furthermore, they showed the propagation of aggregates in the mouse brain by observing, 60 days after O-α-syn injection into the striatum, transmission to the midbrain, frontal lobe and hippocampus of the mouse. Thus, in addition to providing evidence of the toxicity of O-α-syn, the authors suggest the existence of a prion-like propagation mechanism [120].

Tau

Recent experimental evidence has pointed out the importance of extracellular tau oligomers in neurodegeneration, even if the mechanisms by which these aggregates induce cell death are not yet fully understood [121].

It has been reported that oligomers of the 1N4R isoform of phosphorylated tau protein (p-tau1N4R) significantly reduce the number of neurons in primary neuronal–glial cultures prepared from rat cerebellum [122] and, in fact, have been indicated as a relevant marker for the early diagnosis of tauopathies [58]. Similarly, Flach et al., detected, by MTT assay, the reduction in viability of SH-SY5Y cells incubated with pre-aggregated tau protein samples. Specifically, the authors observed the greatest reduction in viability with samples aggregated for a time interval of 6 to 48 h, which corresponds to the formation of toxic oligomers. Increasing the pre-aggregation time of the sample, cell viability increased slightly but did not reach the control levels observed in cells treated with non-aggregated tau [59]. The authors attributed the loss of viability to the compromised membrane integrity due to pore formation. Indeed, they monitored the release of fluorescent-labeled artificial phospholipid vesicles with pre-aggregated tau for 0, 24 and 144 h, detecting maximum vesicular loss with aggregated protein for 24 h. In contrast, samples of non-aggregated and 144 h aggregated tau did not induce vesicular leakage, demonstrating that only oligomeric species are able to permeabilize the membrane [59].

The direct cause of the toxicity of extracellular tau oligomers seemed not to be the hyperphosphorylation of the protein [123] but an electrostatic interaction between the oligomers and the membrane followed by entrainment within the bilayer of the small spherical oligomers [124]. A subsequent conformational transition could cause exposure of the hydrophobic fragments and organization into pore-like structures, although tau-protein-induced amyloid pore formation has not yet been well characterized [124].

Htt

In 2013, Burke et al., reported that the first 17 N-terminal residues of Htt have lipid-binding properties and thus confer huntingtin the ability to interact with a wide variety of membrane-containing structures, and that this interaction further modulates Htt aggregation. Specifically, the authors reported that prefibrillar aggregates of Htt may determine the pathogenesis of HD disease through direct binding to cell membranes, altering their integrity [125]. This agrees with Arndt et al., who in 2015 suggested that there is a correlation between the first 17 Htt residues and the interaction with cellular and subcellular compartments. In particular, the authors suggested that Htt aggregates are able to bind membranes and alter their structure and stability, exerting a cytotoxic action [126].

Furthermore, to strengthen this hypothesis in the pathogenesis of HD, Sedighi et al., recently reported that “sumoylation”, a post-translational modification consisting of the covalent and reversible addition of small ubiquitin-like modifier (SUMO) proteins, prevents the accumulation of Htt aggregates on model lipid bilayers [127].

PrP

In their review, Huang et al., reported that β-sheet-rich PrPSc oligomers are the most neurotoxic species both in vivo and in vitro, suggesting these species as potential drug targets to combat prion diseases [68].

In 2010, Chich et al., studied the interaction of α-helix-rich monomers and β-sheet-rich oligomers with lipid membranes. The two species were separated by SEC and their propensity to permeabilize membranes was studied. Interestingly, the authors observed that while monomers did not alter the properties of lipid membranes, oligomers composed of 12 subunits (dodecamer) significantly increased their permeability. Notably, the authors suggest that dodecamers might act by forming pore-like structures in the membrane and that this event might represent the origin of the neurotoxic mechanism induced by PrP oligomers [128].

In 2011, Sanghera et al., demonstrated that binding of recombinant PrP to membrane “lipid-raft” patterns requires the presence of GM1 [129].

Recently, Huin et al., evaluated the viability of differentiated cerebellar granule cells lacking PrPC, exposed to PrP oligomers. Again, the authors observed a significant rate of neuronal death associated with the ability of the oligomers to permeabilize membranes [130].

#### 3.1.2. Proteins Non-Linked to Neurodegenerative Diseases

HEWL

In 2007, Vieira et al., observed that soluble HEWL oligomers, formed in the early stages of aggregation, were toxic to cultured cortical neurons, inducing hyperphosphorylation of tau protein and stimulating marked neurodegeneration when injected into rat brain [73]. Hyperphosphorylation of tau protein, an important feature in the AD pathogenesis, has recently been shown to be induced by soluble Aβ oligomers [131], suggesting the existence of a common structural component capable of triggering neuronal tau hyperphosphorylation in both Aβ and HEWL oligomers. Furthermore, Meratan et al., observed, through fluorimetric and luminometric assays, the release of mitochondrial enzymes in a concentration-dependent manner due to HEWL oligomers, but not monomers or fibrils [74].

In 2017, Roqanian et al., investigated the possible effects of three different polyphenols on mitochondrial membrane permeabilization induced by HEWL oligomers. By monitoring the release of mitochondrial enzymes, such as malate dehydrogenase (MDH), the authors reported that HEWL prefibrillar aggregates, but not monomers and fibrils, were able to interact with the membrane by increasing its permeabilization. Interestingly, the authors reported that the three tested polyphenols, curcumin, quercetin and resveratrol, exerted protective effects against the toxic action induced by HEWL oligomers by counteracting mitochondrial membrane permeabilization [132].

Insulin

In 2012, Kachoocei et al., with the aim of investigating the cytotoxicity of different insulin aggregates, evaluated the morphological changes in differentiated PC12 cells. They administered insulin to the cells in oligomeric, protofibrillar and fibrillar forms, assessing parameters such as cell body area, neurite length and width. The authors noted a significant increase in cell body area in cells treated with all three species, in particularly with oligomers. In addition, the length of the neurites was significantly reduced while their width dramatically increased. In addition, cells exposed to treatment with insulin aggregates showed a reduction in both the number of primary neurites and of branching nodes, suggesting that insulin aggregates compromised the complexity of PC12 cells. Although the authors noted a cytotoxic effect induced by major aggregates, they observed that the effects caused by aggregates appeared to be reduced in the more ordered species, suggesting that the formation of MFs, which showed reduced neurotoxicity compared to oligomers, may be a protective mechanism [133].

In 2017, Iannuzzi et al., studying the effects of vanillin on amyloid aggregation, assessed (MTT assay) the viability of SH-SY5Y cells exposed to insulin aggregates. They observed that cells exposed to aggregated insulin samples for 12 h, corresponding to PFOs, reduced cell survival by approximately 45%, whereas prolonged incubation (24 h), which produces fibrillar aggregates, did not affect cell viability. In addition, the authors reported that vanillin strongly influenced insulin aggregation, accelerating the formation of harmless fibrils and stabilizing amyloid aggregates in a non-toxic state [134].

Recently, Sirangelo et al., to investigate the anti-amyloidogenic properties of hydroxytyrosol (HT), an important component of olive oil, evaluated the effects of insulin aggregates on the cell viability of SH-SY5Y cells. The authors observed a 50% reduction in the cell survival of cells treated with insulin oligomeric aggregates, compared with untreated cells, while no toxicity was induced by amyloid fibrils. Surprisingly, samples of aggregated insulin in the presence of HT were not able to induce cytotoxicity, demonstrating that HT may counteract insulin aggregation and thus its toxic action [135].

Sup35

In 2011, Liu et al., compared the cytotoxicity of Sup35 prefibrillar aggregates and fibrils using a cell viability assay. They reported that prefibrillar aggregates significantly reduced the viability of African green monkey kidney cells (Vero cells), whereas MFs were not cytotoxic [85].

In 2012, Krishnan et al., examined the characteristics of Sup35 aggregation intermediates and reported that oligomers formed in the early aggregation stages, which react with the A11 antibody, were toxic to neuronal cells. Specifically, these aggregates were incubated with SH-SY5Y cells and toxicity was assessed by staining with propidium iodide, monitoring the release of adenylate kinase (AK). The authors reported that oligomers capable of reacting with the A11 antibody were highly toxic, while larger aggregates and monomers did not significantly increase AK release [136].

In the same year, Bucciantini et al., demonstrated the importance of GM1 ganglioside in driving Sup35 aggregate-induced cytotoxicity. In particular, they reported that removal of negatively charged sialic acid from amphipathic GM1 molecules by neuraminidase (NAA) treatment effectively counteracted the toxic effects of Sup35 aggregates in murine endothelioma cells (H-END) [137].

In a more recent report, Bucciantini et al., investigated the importance of GM1 in the mechanism of action of Sup35 aggregates, suggesting that the cytotoxicity of amyloid aggregates may depend not only on their structural characteristics, but also on the physico-chemical properties of the interacting molecules. Notably, they commented that “the interactions of oligomers with membrane components, such as anionic lipids, may be non-specific and mediated by covalent, non-covalent, ionic or hydrophobic bonds” [97].

PI3-SH3

In 2002, Bucciantini et al., measured the cell survival (MTT assay) of NIH-3T3 cells exposed to different PI3-SH3 aggregates. Treatment with highly structured fibrils did not change cell viability, whereas the application of granular aggregates, produced by a short incubation period, significantly reduced NIH-3T3 cell survival. The authors reported that prefibrillar aggregates of PI3-SH3 can be cytotoxic, surprisingly, reproducing the cell deterioration effects of Aβ42 aggregates [19].

HypF-N

In 2018, Oropesa-Nuñez et al., evaluated the effects of the interaction of two types of HypF-N oligomers, similar in morphological characteristics but different in cytotoxicity, with the plasma membrane of Chinese hamster ovary cells [138]. Specifically, they calculated the work required to separate the oligomers from membranes, observing that the most toxic oligomers bound to the membranes much more efficiently than the non-toxic species. In addition, the authors studied which components of the plasma membrane contributed most to the efficiency of oligomer–membrane binding. By treatment with neuraminidase, they identified GM1 as a major mediator of oligomer–membrane interaction and associated cytotoxicity [138]. More recently, Farrugia et al., studying the damaging effects of two types of HypF-N oligomers on mitochondrial membranes, reported that oligomers characterized by high surface hydrophobicity caused a strong permeabilization of mitochondrial liposomes, which resulted in a lowering of membrane potential, cytochrome c release and mitochondrial dysfunction. In contrast, oligomers characterized by a low surface hydrophobicity were not able to permeabilize membranes, suggesting a correlation between the surface exposure of hydrophobic regions and oligomer toxicity [139]. This is in agreement with the study performed by Capitini et al., on the structural differences between toxic and non-toxic HypF-N oligomers, according to which the formation of a highly organized core in toxic oligomers results in the exposure of more hydrophobic residues than in non-toxic species, thus promoting aberrant interaction with cellular components [140].

In 2012, Evangelisti and colleagues evaluated the effects of a membrane modification on the cytotoxicity of HypF-N oligomers in SH-SY5Y cells [141]. Using Hoechst staining and MTT assay, it was shown that membrane composition influenced the toxicity of HypF-N oligomers. Specifically, cell viability was preserved either by an increase in cholesterol content, which induced alterations in structural stiffness, or by a reduction in GM1 sialic acid content, which was responsible for alterations in charge density. Notably, the authors reported that GM1 levels played a dominant role in the cytotoxicity of HypF-N oligomers, which is expressed through an extracellular Ca^2+^ influx. These results suggest that amyloid cytotoxicity depends on two factors: i) the specific properties of aberrant protein groups, such as disorder, flexibility and surface exposure of hydrophobic groups; and ii) the physicochemical characteristics of membranes, which depend on lipid composition, such as stiffness and electrostatic potential [141].

sCT

In 2014, we demonstrated that sCT PFOs, but not monomers or other types of aggregate (APFs, LPFs or MFs), induced a strong Ca^2+^ influx in mature hippocampal neurons, causing apoptosis and neurotoxicity [92]. We hypothesized that the action of sCT PFOs might be mediated by the formation of “amyloid pores” as a result of their interaction with LRs in membranes [142]. Our data suggest that the toxicity of sCT oligomers depends on two structural factors: (i) their surface charge, positive at physiological pH, and (ii) their partially ordered, flexible structure with an external amphipathic profile [93]. LRs contained in membranes represent the natural target of sCT PFOs, as we observed that this interaction is favored in the presence of negatively charged molecules, especially GM1 and cholesterol [143].

In 2019, we evaluated the effects of sCT PFOs in primary neurons and mouse hippocampal slices in terms of Ca^2+^ influx, cell viability, LTP impairment, post-synaptic densities (PSD) and synaptophysin expression. Our results indicate that sCT oligomers induced an abnormal Ca^2+^ influx, partly attributable to the NMDA receptor’s (NMDAR) activation. Thus, we proposed an innovative “unified” neurotoxicity mechanism for amyloid proteins, according to which membrane permeabilization by itself is not sufficient to trigger neurotoxicity, but can nevertheless induce abnormal activation of NMDAR [25]. Notably, these results are very similar to those obtained for amyloid proteins, such as Aβ [109].

Very recently, we studied in cellular models and in mouse hippocampal slices the protective effect exerted by NAA, an enzyme able to cut the negatively charged sialic acid present in the GM1 of the neuronal membranes, from the neurotoxicity induced by sCT PFOs. Notably, we were able to totally inhibit the neurotoxicity in both models, demonstrating the importance of the electrostatic interaction in the molecular mechanisms controlling the detrimental effects of amyloid PFOs [144].

### 3.2. Synaptic Effects

Impairment of synaptic function is a common occurrence in all neurodegenerative diseases. Indeed, it is known that in the presence of PFOs, synaptic protein expression, as an effect of the toxicity, and synaptic plasticity, due to the impairment of vesicles recycle, were observed [145,146,147,148].

#### 3.2.1. Synaptotoxicity

We summarize the main information in Table 1, focusing on the principal synaptic proteins involved in amyloid toxicity and the effects caused by their altered expression.

Aβ

Defects in the fine-tuning of synaptic vesicle dynamics at the presynaptic level by Aβ oligomers impair synaptic homeostasis and contribute to synaptic loss. The level of SNARE complex formation and SNARE-mediated exocytosis in Aβ PP-PS1 mice is significantly reduced [159]. In addition, Aβ oligomers reduce the efficacy of synaptic vesicle recycling and alter the recycling/resting pool ratio by expanding the resting fraction at the expense of the recycling fraction [160]. Moreover, a decrease in synaptophysin was observed in AD animal models [161].

To determine how early synapse loss occurs in AD, Hong et al., performed a super-resolution structured illumination microscopy (SIM) study, quantifying synapse density in the hippocampal CA1 radiatum stratum of familial AD-mutant hAPP (“J20”) transgenic mice [149]. A significant reduction in synaptophysin and post-synaptic density protein 95 (PSD-95) was found, resulting in synapse loss in the J20 hippocampus at 3–4 months old, an age prior to plaque deposition. This synaptic loss, which characterizes the early phase of AD, appears to be mediated by inappropriate activation of complement and microglia, leading to neuroinflammation and cognitive decline [149]. Similarly, Manczak and colleagues observed that the hippocampus of 12-month-old APP transgenic mice was characterized by low levels of synaptophysin and PSD-95, as well as a significant reduction in microtubule-associated protein 2 (MAP2), a protein essential for dendritic growth, suggesting that reduced synaptic proteins and decreased dendritic spine density are undoubtedly responsible for synaptic damage and cognitive decline in APP mice [150].

Cytoskeletal remodeling that induces alterations, such as axon and dendrite degeneration and the development of dystrophic neurites and tau phosphorylation, are reported as hallmarks of AD neurodegeneration and are also involved in the loss of synapses and dendritic spines [162].

α-Syn

Incubation of primary hippocampal neurons with α-syn oligomers has been shown to cause synaptotoxic failure, primarily due to a decrease in synaptic vesicle protein 2 (SV2), a membrane protein of synaptic vesicles associated with neurotransmitter release [153]. In an E57K-mutant mouse model, which is inclined to form oligomeric α-syn, loss of synapses and dendrites as well as decreased levels of synapsin 1 and synaptic vesicles were observed, and these led to further behavioral defects [163].

Furthermore, quantitative immunofluorescent confocal microscopy analysis performed on primary hippocampal neurons treated with α-syn oligomers revealed increased staining for the synaptosomal-associated protein 25 kDa (SNAP25), which is responsible for the association between synaptic vesicles and membranes [153]. Therefore, it was proposed that α-syn oligomers, by forming a pore in hippocampal neuronal membranes, induce an increase in intracellular Ca2+ influx, which in turn is responsible for vesicular depletion and synaptotoxicity [153].

Tau

It has been suggested that changes in tau protein acetylation may represent an early event that can induce aggregation and phosphorylation, resulting in cognitive impairment [164,165]. Furthermore, it has been reported that tau oligomers can impair presynaptic function through binding to synaptogyrin-3, a vesicular transmembrane protein [151], and that tau accumulation at the presynaptic level results in vesicular depletion and synaptic depression [166,167]. To assess the synaptic effect of tau protein at different aggregation states, Lasagna-Reeves et al., evaluated the expression of several proteins associated with synaptic function, such as synaptophysin, synapsin-1 and septin-11, in a mouse hippocampal homogenate. It was found that synaptophysin levels were significantly lower in tissues treated with tau oligomers than in those treated with fibrils or monomers [152]. Similarly, septin-11 levels were also significantly reduced in brains injected with tau oligomers, whereas no differences were found in synapsin-1 analysis. Furthermore, immunohistochemistry performed in the CA1 region of the brain hemisphere treated with tau oligomers showed a marked reduction in synaptophysin density, whereas sections treated with monomers and fibrils showed no changes in staining [152]. Based on this evidence, synaptic dysfunction and abnormalities in axonal transport are probably the early pathogenic events in tauopathies, which precede the formation of neurofibrillary tangles and subsequent neuronal cell death.

Htt

The Htt protein can interact with a wide variety of pre- and post-synaptic proteins [168], as well as being involved in vesicular trafficking [169]. In this regard, Morton et al., found a progressive loss of presynaptic protein complexin II and synaptobrevin 2, a small vesicular transmembrane protein, both in the brains of R6/2 mice transgenic for the HD mutation and in the striatum of post-mortem brains of HD patients [154]. These results suggest that abnormalities observed in the expression of proteins known to be involved in the control of neurotransmitter release, including both modulators and major components of the vesicle fusion mechanism, could explain at least some of the functional abnormalities observed in HD [154]. In agreement with these data, Smith and colleagues, investigating the expression levels of several synaptic proteins essential for neurotransmitter release, observed that a decrease in SNAP25 protein levels is always accompanied by a loss of rhabphilin 3a in patients with a higher pathological degree of disease [155]. In addition, Shirendeb et al., evaluated the effects of mutant Htt on synaptophysin and MAP2 levels by immunostaining 10 DIV neurons from BACHD mice, i.e., transgenic mice expressing the human HTT gene with 97 repeats. A significant reduction in synaptophysin and MAP2 immunoreactivity was reported compared to WT neurons, indicating that the mutated form of Htt promotes synaptic dysfunction [156].

PrP

Synaptic dysfunction is also a feature of prion diseases [170]. Belichenko et al., using two different mouse models of scrapie, detected the presence of a pre- and post-synaptic site alteration, which could be responsible for the disruption of neuronal circuits and the initiation of an apoptotic process, thus giving rise to the neurological disorders that characterize prion diseases [171]. Indeed, it has been reported that synaptic dysfunction precedes neuronal loss; however, it is unclear whether, and through which mechanisms, the disruption of synaptic activity ultimately leads to neuronal death. In this regard, Ghirardini et al., have recently demonstrated that mutant PrP alters the secretory trafficking of alpha-amino-3-hydroxy-5-methyl-4-isoxazolepropionic acid receptors (AMPAR) [157]. Specifically, it has been reported that intracellular retention of the GluA2 subunit causes synaptic exposure of GluA2-deficient and Ca^2+^-permeable AMPAR, resulting in increased Ca^2+^ permeability and enhanced sensitivity to excitotoxic cell death [157]. Thus, it is possible that AMPAR represent pathogenic targets in prion diseases, confirming the involvement of excitotoxicity in neurodegeneration.

Cunningham and colleagues performed synaptophysin immunostaining to obtain information on synaptic density in the hippocampus of mice infected with scrapie ME7 [158]. Thirteen weeks post-infection, loss of synaptophysin staining was found in the radiate layer. In addition, synaptophysin staining was also progressively reduced in the proximal dendrite region of CA1 pyramidal cells until the nineteenth week after infection [158]. These findings provide a further step forward in understanding the neuropathogenesis of prion diseases.

sCT

Consistent with its amyloid nature, sCT oligomers also induce cellular damage very similar to that caused by Aβ oligomers. Indeed, it has been reported that treatment of primary hippocampal neurons with sCT oligomers caused deterioration of the dendritic tree, loss of the finest branches and synaptotoxicity, as evidenced by reduced expression of MAP2 and synaptophysin and subsequent loss and/or alteration of synaptic structures [143]. These results are in agreement with data obtained from Western blot and immunofluorescence analyses performed on mouse hippocampal slices perfused with sCT oligomers, in which we also observed a significant reduction in PSD-95, suggesting that synaptic damage can be considered as one of the main contributors to neuronal dysfunction related to cognitive impairment [25].

#### 3.2.2. Synaptic Plasticity

**Figure 3:**
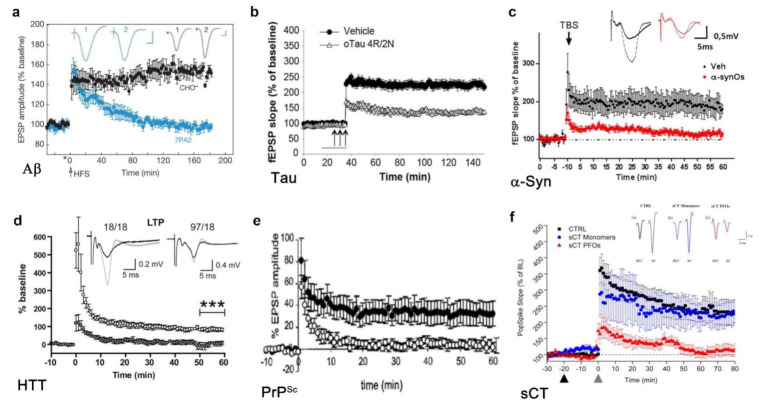
Reduction in LTP observed in models for the study of the amyloid neurotoxicity. (**a**) Alzheimer’s disease: microinjection of Aβ oligomers (blue) strongly reduced hippocampal LTP compared to control (black) (with the courtesy of Walsh et al. [178]); (**b**) Alzheimer’s disease: perfusion with tau oligomers (white) impairs LTP in mouse hippocampal slices (with the courtesy of Fá et al. [179]); (**c**) Parkinson’s disease: α-syn oligomers (red) reduce LTP in the CA1 region in mouse hippocampal slices (with the courtesy of La Vitola et al. [180]); (**d**) Huntington’s disease: hippocampal LTP recorded in the CA1 region of hippocampal slices from 9-month-old Hu97/18 model mice (black) is significantly reduced compared with that of animals of the same age (white) (with the courtesy of Kolodziejczyk et al. [177]); (**e**) prion disease: mice infected with scrapie ME7 shows a strongly reduced LTP in the CA1 area (white) (with the courtesy of Johnston et al. [181]); (**f**) amyloid model: treatment with sCT PFOs (red) results in impairment of LTP in mouse hippocampal slices while slices treated with monomeric sCT (blue) show no significant changes compared to control (black) (with the courtesy of Belfiore et al. [25]).

The pathogenesis of amyloid diseases could be explained by a loss of plasticity that can negatively affect dendritic branching, synaptic remodeling, LTP, axonal sprouting and neurite extension, as well as synaptogenesis and neurogenesis processes [168,172,173,174]. Indeed, plasticity, the process by which synapses modulate their strength and form new neuronal connections, is known to play an essential role in response to injury and disease [175]. Moreover, most of the scientific evidence in the literature agrees that the synaptic plasticity loss is induced by PFOs [22,23,24,25,176,177], as shown in Figure 3. For this reason, electrophysiological recordings of excitatory field potentials could represent an excellent strategy to assess the synaptotoxic effects of amyloid oligomeric aggregates.

Aβ

It has been widely documented that hippocampal LTP, a measure of synaptic plasticity, is particularly sensitive to the toxic action of Aβ oligomers [22,182,183]. In this regard, Walsh et al., demonstrated that brain microinjection of Aβ oligomers, obtained from 7PA2 Chinese hamster ovary (CHO) cells expressing the V717F AD mutation in APP751, markedly inhibited hippocampal LTP of rats in vivo, whereas their immunodepletion from the medium completely abrogated this effect [178]. Similarly, Li and colleagues observed that inhibition of hippocampal LTP and subsequent loss of synapses and neurites was caused not only by soluble Aβ oligomers, but also by oligomers isolated directly from the cerebral cortex of AD patients [184]. A strong abrogation of LTP has also been achieved using synthetic Aβ oligomers, although higher neurotoxicity has been reported for natural Aβ forms [185]. Furthermore, Aβ oligomers have been observed to promote the phenomenon of long-term depression (LTD) in the hippocampus, characterized by a reduction in the volume and number of dendritic spines, similar to what occurs in AD patients’ brains [185].

The synaptotoxic effects of Aβ oligomers would appear to depend on their interaction with membrane receptors, resulting in an alteration of the molecular pathways involved in neuronal functions responsible for the transmission and storage of information in the brain. Acute exposure to Aβ oligomers impairs LTP in rodent tissue, predominantly by acting at the post-synaptic compartment [186].

According to most scientists, the synaptotoxicity induced by Aβ oligomers depends on both the activation of metabotropic glutamate receptor type 5 (mGluR5) and the stimulation of three kinases (c-Jun N-terminal protein kinase 1, JNK1; cyclin-dependent kinase 5, Cdk5; p38 mitogen-activated protein kinase, p38 MAPK), which are responsible for LTP inhibition [187]. Furthermore, a role for protein phosphatase 1 (PP1) as an important player in the mechanisms of Aβ oligomer-induced toxicity has been suggested. In this regard, it has been observed that suppression of the PP1 gene in arcAβ mice, which express the APP protein with Swedish and Arctic mutations conferring an increased propensity to form oligomers, promotes LTP preservation [188]. Finally, it has been suggested that initial synaptic dysfunction, possibly followed by synapse loss, may result from the excessive activation of NMDA receptors, which are extremely sensitive to the synaptotoxic action of Aβ oligomers [189,190].

Tau

The effects of tau oligomers on neuronal properties have been investigated through several electrophysiological studies involving extracellular application of these toxic species in mouse models of transgenic tauopathy, in which mutant forms of the tau protein, which tend to aggregate, are overexpressed [147,191,192]. Oligomeric tau has been shown to alter the intrinsic excitability of neurons and modulate short- and long-term plasticity [176]. In particular, Ondrejcak et al., demonstrated that the administration by intracerebroventricular injection of recombinant aggregated tau protein, or tau protein isolated from AD patients, inhibited hippocampal LTP, suggesting a post-synaptic action for tau oligomers [193]. In agreement with these observations, Fà and colleagues found that brief exposure to extracellular recombinant human tau oligomers, but not to monomers, resulted in impaired LTP and memory [179]. In addition, the simultaneous presence of tau and Aβ oligomers at sub-toxic doses would appear to inhibit LTP, suggesting cooperation of the two species in the onset of the typical features of AD [179]. The effective involvement of tau protein in the impairment of LTP by Aβ oligomers was investigated by Shipton et al., who, using tau protein (tau-/-) knock-out mice, showed that the LTP inhibition at the CA3–CA1 synapse induced by Aβ oligomers could be counteracted by the absence of tau, underlining tau’s role as a mediator of Aβ-induced synaptotoxicity [194]. Further confirmation was recently provided by Ruan and colleagues, who demonstrated that tau oligomers extracted from the cerebral cortex of AD patients strongly inhibited LTP in mouse hippocampal slices and impaired memory in wild-type mice, confirming the ability of such soluble species to induce the typical symptoms of neurodegeneration [195].

α-Syn

The synaptic mechanisms underlying the behavioral and motor effects induced by the presence of α-syn oligomers are still unclear. Tozzi et al., found that overexpression of truncated or wild-type human α-syn caused a partial reduction in striatal dopamine levels and selectively blocked LTP induction in striatal cholinergic interneurons, producing early mnemonic and motor alterations [196]. These effects depended on α-syn modulation of the GluN2D-expressing NMDA receptors in cholinergic interneurons, suggesting that striatal cholinergic dysfunction, induced by a direct interaction between α-syn oligomers and NMDA receptors, is an early biological marker of the disease [196].

Similarly, electrophysiological recordings performed on hippocampal slices treated with α-syn oligomers revealed inhibition of hippocampal LTP [180]. Furthermore, mice overexpressing mutated human α-syn showed, in granule cells of the hippocampal dentate gyrus, the onset of LTD after high-frequency stimulation, suggesting that overexpression of a mutated form of α-syn may intensify the aging process and alter hippocampal synaptic plasticity [197]. Notably, it was reported that α-syn oligomers, but not monomers and fibrils, significantly impaired hippocampal LTP recorded in the CA1 region [23]. Moreover, Diógenes et al., observed that prolonged exposure to α-syn oligomers increased basal synaptic transmission through NMDA receptor activation, triggering an increased contribution of Ca2+-permeable AMPAR [23].

Htt

Although there are few data on the effect of Htt oligomers on synaptic transmission, mutations caused by trinucleotide repeat expansion (CAG) in the HTT gene are known to promote cognitive deficits and synaptic loss [198]. These disorders have been shown to be accompanied by important electrophysiological changes; in fact, field potential values recorded in the CA1 region of hippocampal slices revealed a complete absence of LTP, indicative of spatial learning deficits [177]. Similar results were also observed in the R6/2 transgenic mouse model, in which impaired transmission and synaptic plasticity, as well as spatial cognition, were found even before neuronal loss [199]. Finally, immunopurification with the A11 antibody specific for Htt oligomers, performed on brain homogenates from two HD mouse models (R6/2 and YAC128), showed the presence of oligomeric species with seeding properties, suggesting an involvement of these species in the synaptic transmission deficits that characterize HD [66].

PrP

It was reported that scrapie-infected mice showed altered synaptic activity in the hippocampal CA1 area; specifically, excitatory post-synaptic field potentials were not altered in the early stages of the disease, while at a more advanced stage, a significant change in the ability of hippocampal slices to maintain LTP was observed [181]. This suggests that the conversion of the cellular prion protein (PrPC) to the scrapie, protease-resistant form of PrP (PrPSc) may be responsible for the impairment of the mechanisms that stabilize the LTP maintenance phase [181].

Recently, it was observed that treatment for 5 min with oligomers isolated from the brains of mice dying of M1000 prion disease significantly reduced LTP in the CA1 region of mouse hippocampal slices [24]. Intra-hippocampal injections of a scrapie-infected cell homogenate have also been reported to cause cognitive impairment in mice, probably related to alterations in synaptic function [200]. Indeed, in ME7 scrapie-infected mouse, both a loss of the perineuronal CA1 subfield network and a loss of the hippocampal subiculum were found, both of which correlated with electrophysiological and behavioral alterations, suggesting that the degeneration and subsequent impairment of synaptic plasticity may depend on the extracellular accumulation of a soluble protein intermediate of PrPSc [201]. Indeed, the onset of synaptic plasticity abnormalities, an early symptom of the disease, coincides with the deposition of PrPSc [202].

sCT

In our previous work, we evaluated the neurotoxic effects of sCT oligomers on mouse hippocampal slices using in vitro electrophysiological recordings [25]. Our results show that perfusion of the slices, for twenty minutes, with artificial cerebrospinal fluid (ACSF) solution enriched in sCT oligomers was able to strongly depress the LTP recorded in the hippocampal CA1 subfield [25]. Indeed, PS amplitude values were significantly lower than those of the control groups and, eighty minutes after high-frequency stimulation, returned to values similar to those of basal synaptic transmission. The same synaptotoxic action was not obtained with the monomeric form of calcitonin, confirming that oligomeric aggregates are the species with the greatest neurotoxic power [25]. Notably, the LTP reduction caused by sCT oligomers was very similar to that reported for Aβ oligomers, suggesting the existence of a common mechanism of neurotoxicity [178]. Based on these findings, we hypothesized that the interaction of sCT oligomers with the cell membrane causes abnormal calcium influx, which in turn is responsible for impaired synaptic transmission and vesicle depletion, resulting in synapse silencing [25].

## 4. Conclusions

Amyloid-associated neurodegenerative diseases are characterized by intra- or extracellular accumulation of specific proteins in the CNS. Although these proteins differ in their primary sequence, they all share the tendency to adopt an incorrect conformation and the propensity to aggregate, starting from the formation of soluble low-molecular-weight PFOs to insoluble aggregates such as MFs. Interestingly, the ability to create soluble PFOs has also been observed in proteins non-linked to neurodegenerative diseases. Thus, given the metastable nature of amyloid oligomers and the extremely heterogeneous nature of the aggregation process, it is possible to use these proteins as models to reproduce the effects of amyloid in vitro.

It has been proposed that PFOs, considered the most neurotoxic of all aggregates, share a “common structure” and a “common mechanism” by which they induce neuronal damage and death. Thus, our interest focused on the structure and activity of this type of amyloid structure.

**Figure 4:**
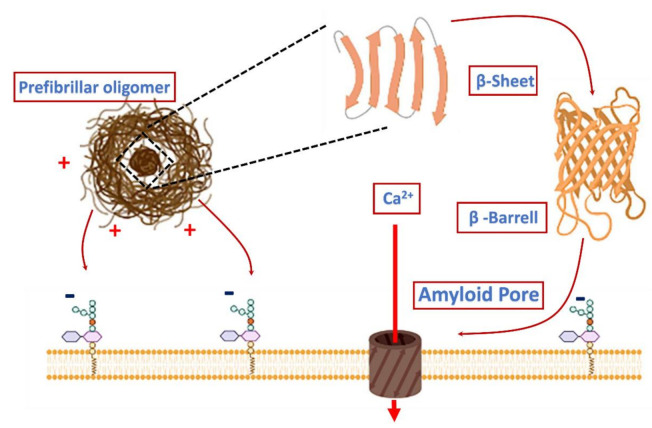
The possible pore formation mechanism by an amyloid PFO. According to the most recent data collected in the literature, the typical amyloid PFO may have the shape of a sphere with a stiff hydrophobic core, partially structured in a β-sheet. The surface of the sphere exposes both polar and hydrophobic protein sequences. The first, exposed to the solvent and likely charged, are responsible for interaction with charged parts of the membranes, such as the GM1 forming the “lipid-rafts”. After the contact, the partially ordered PFO hydrophobic core, in order to minimize the hydrophobic mismatch with the membrane backbone, forms a permeable amyloid pore assuming a β-barrel conformation favored by the ordered structure of the “lipid-rafts”.

Concerning the PFOs structure, only a few molecular resolution studies have been performed so far on several proteins. However, in our opinion, some commonalities can be noted. Firstly, they are all small globules ranging in diameter from 5 to 10 nm, formed by low-molecular-weight oligomers, typically from tetramers to dodecamers. This morphology maximizes the aggregate surface/volume ratio, favoring its interaction with the cellular membranes. Their structure is generally metastable, characterized by many proteins in the random configuration, and an internal core in a more ordered but not-crystalline configuration, typically a β-sheet. The hydrophobic parts of the proteins generally form the hydrophobic core and the N- and C-terminal parts are oriented outside and exposed to solvent. In this arrangement, the oligomer surface is made of flexible and disordered domains that expose the hydrophilic protein parts to the solvent, together with stiff and more ordered hydrophobic patches only partially accessible to the solvent. Recently, it has been proposed that the electrostatic interaction between the flexible and hydrophilic protein parts of the PFOs and the charged parts of the “lipid-rafts” in the membrane drives the binding of PFOs to the target neurons (Figure 4). Notably, disordered aggregates lacking the core, or totally ordered aggregates such as spherical protofibrils, were unable to induce detrimental effects.

Concerning the PFOs’ neurotoxicity mechanisms, it has been proposed that a common core of pathologic pathways exists for amyloid-associated diseases, based on the cellular membrane permeabilization and the subsequent abnormal Ca^2+^ influx, induced by aggregates of the involved proteins independently from their primary sequence.

The intriguing hypothesis has been formulated that amyloid diseases are caused by aggregates that mimic bacterial pore-forming toxins, which in general form well-ordered oligomeric membrane-spanning pores. Indeed, it is generally accepted that PFOs are characterized by a structure that promotes their insertion into biological membranes, resulting in the formation of a non-selective permeable pore: the “amyloid pore”. Notably, several biophysical and computational studies indicate that the PFO structural arrangement, described before, favors its insertion in the biological membranes.

Many studies indicated that the neuronal membrane composition and its chemical microenvironment also play a pivotal role. In fact, it was shown that the brains of AD patients contained increased fractions of anionic lipids and that anionic lipids and not neutral lipids favored cationic influx. It is now generally accepted that “lipid-rafts”, which are ordered nanodomains formed by sphingolipids and cholesterol abundant in the outer leaflet of the plasma membrane, play a special role.

However, it has been demonstrated that the process of “amyloid pore” formation is not able to induce neurotoxicity per se, and that the involvement of ionotropic glutamate receptor NMDA, the uncontrolled activation of which promotes an abnormal Ca^2+^ influx, is necessary. The increase in the intracellular Ca^2+^ concentration is the key event that triggers the processes leading to cell death. The reserve pool of synaptic vesicles containing the neurotransmitter is depleted; in fact, protein levels in the vesicular membranes are drastically reduced. Subsequently, there is the impairment of post-synaptic structures, as evidenced by the reduced expression of proteins typical of dendritic spines.

**Figure 5:**
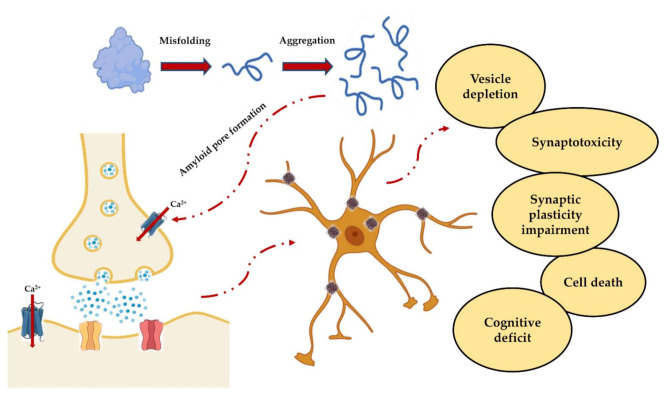
Schematic representation of the events leading to neuronal death and subsequent cognitive impairment. Misfolding and aggregation lead to the formation of PFOs, which not only result in the formation of insoluble fibers that are deposited in the nerve tissue, but also form amyloid pores in the synaptic membrane. The resulting deregulation of the Ca^2+^ homeostasis is responsible for the cascade of events that include vesicular depletion, synaptotoxicity, impaired synaptic plasticity and, ultimately, cell death and cognitive decline.

This generalized scenario, summarized in Figure 5, leads to the functional deterioration that is observed in vitro with the impairment of transmission and synaptic plasticity, while in vivo with progressive cognitive deterioration.

## Figures and Tables

**Table 1:** Main synaptic proteins affected by the action of amyloid aggregates and the consequent neurodegenerative effects.

Amyloid Oligomers	Proteins Involved	Synaptic Effects	Neurodegenerative Disease	References
**A*β***	Synaptophysin	Vesicular depletion	AD	[149]
	PSD-95	Decrease in dendritic spine density		[149,150]
	MAP2	Dendritic tree reduction		[150]
**Tau**	Synaptogyrin-3	Impair presynaptic function	AD and tauopathies	[151]
	Synaptophysin Septina-11	Impaired presynaptic density and neuronal trafficking deficit		[152]
***α*-syn**	SV2 SNAP25	Alteration of both neurotransmitter release and vesicle–membrane fusion	PD	[153]
**HTT**	Complexin II Synaptobrevin 2	Altered neurotransmitter release	HD	[154]
	SNAP25 Rhabphilin 3a	Vesicle–membrane fusion and vesicle recycling deficit		[155]
	Synaptophysin MAP2	Vesicular depletion and reduction in dendritic spine density		[156]
**PrP**	GluR2 subunit of AMPAR	Increased permeability to Ca^2+^	Prion’s disease	[157]
	Synaptophysin	Vesicular depletion		[158]
**sCT**	Synaptophysin MAP2	Vesicular depletion and dendritic tree alteration	No neurodegenerative disease	[143]
	PSD95	Decrease in dendritic spine density		[25]

Aβ:amyloid-β; α-syn: α-synuclein; HTT: huntingtin protein; PrP: prion protein; sCT: salmon calcitonin; PSD-95: post-synaptic Density Protein-95; MAP2: microtubule-associated protein 2; SV2: synaptic vesicle protein 2; SNAP25: synaptosomal-associated protein 25 kDa; AD: Alzheimer’s disease; PD: Parkinson’s disease; HD: Huntington’s disease; AMPAR: alpha-amino-3-hydroxy-5-methyl-4-isoxazolepropionic acid receptors.

## Data Availability

No new data were created or analyzed in this study. Data sharing is not applicable to this article.
